# Plasminogen activator inhibitor-1 as an oncologic target: biology, therapeutic inhibitors, and clinical translation

**DOI:** 10.3389/fonc.2026.1737975

**Published:** 2026-04-01

**Authors:** Nada Saed Homod Al Shaer, Aya Tleyjeh, Maryam Alawi Al-Attas, Joudi Haitham Alibrahim, Mohammed Imran Khan, Ahmed Yaqinuddin

**Affiliations:** 1 College of Medicine, Alfaisal University, Riyadh, Saudi Arabia; 2 King Faisal Specialist Hospital and Research Center, Jeddah, Saudi Arabia

**Keywords:** cancer biomarkers, cancer progression, combination therapy, pai-1 inhibitors, plasminogen activator inhibitor-1 (PAI-1), Tumormicroenvironment

## Abstract

Plasminogen activator inhibitor-1 (PAI-1) is a key regulator of cancer biology, influencing tumor progression, metastasis, and therapeutic resistance. Elevated levels of PAI-1 are consistently associated with poor prognosis across several malignancies, including breast, ovarian, lung, hepatobiliary, colorectal, and glioblastoma. Mechanistically, PAI-1 facilitates cancer cell survival, endothelial migration, and immune modulation. From a therapeutic standpoint, PAI-1 represents a promising target. Strategies to inhibit PAI-1, including small-molecule inhibitors and monoclonal antibodies, have demonstrated promise in preclinical research. Collectively, these findings underscore both the therapeutic potential and the translational challenges associated with targeting PAI-1 in future cancer treatment. This review examines the mechanistic foundations of the PAI-1 paradox, its prognostic and therapeutic implications in cancer, and the opportunities and challenges for clinical translation.

## 1 Introduction

Cancer progression and mortality are driven primarily by metastatic dissemination and treatment resistance rather than the primary tumor itself. Despite advances in modern oncology, many solid tumors eventually recur or fail to respond durably to therapy, highlighting the need to better understand biological pathways that regulate tumor invasion and survival.

Tumor cell invasion and metastasis require the coordinated and temporally regulated interplay of adhesion, proteolytic, and migratory mechanisms. Central to these processes is the plasminogen activator (PA)-plasmin system. The serine proteases urokinase-type (uPA) and tissue-type (tPA) plasminogen activators convert inactive plasminogen into plasmin, a broadly acting enzyme capable of degrading extracellular matrix components, activating growth factors, and stimulating metalloproteinases. The binding of plasminogen and uPA to their respective receptors localizes plasmin activity to the tumor cell surface, thereby facilitating migration and invasion. These proteolytic events are tightly regulated by specific inhibitors, plasminogen activator inhibitors 1 and 2 (PAI-1 and PAI-2) (1).

Paradoxically, despite its role as a serine protease inhibitor, elevated PAI-1 expression is consistently associated with poor prognosis in a variety of cancers (1). This apparent contradiction suggests that PAI-1 has functions beyond protease inhibition that influence tumor behavior. Indeed, an extensive body of literature highlights the pro-tumorigenic functions of PAI-1, yet there remains limited clinical evidence that its inhibition provides therapeutic benefit in patients (2). Given the central role of the uPA system in metastatic progression, it has been widely recognized as a promising therapeutic and gene therapy target in oncology (3). Moreover, PAI-1 has emerged as a robust biomarker of cancer prognosis and therapeutic responsiveness. Nonetheless, efforts to target PAI-1 directly remain hindered by the challenge of developing potent and durable inhibitors of its active form that are suitable for chronic clinical use (2).

Despite extensive investigation, the role of PAI-1 in cancer remains unclear. Experimental and clinical observations appear contradictory, with studies reporting both anti-invasive and tumor-promoting effects depending on the biological context. This lack of conceptual clarity has limited translation of PAI-1 biology into therapeutic strategies. This review aims to integrate mechanistic, biological, and translational evidence to clarify the so-called PAI-1 paradox. We discuss how concentration, compartmentalization, and binding state influence the net effects of PAI-1 on tumor invasion, microenvironmental interactions, and therapeutic response. We also evaluate the implications of these findings for biomarker development and the feasibility of targeting PAI-1 in cancer therapy.

## 2 The uPA/uPAR/PAI system in cancer biology

The fibrinolytic system, which involves the generation of plasmin through plasminogen activation, plays a crucial role in extracellular matrix (ECM) degradation and cell signaling through the activities of urokinase-type plasminogen activator (uPA) and its receptor, the urokinase plasminogen activator receptor (uPAR) (4). Its inhibition is mediated primarily by specific serpins, notably plasminogen activator inhibitor-1 (PAI-1), which serves as the main inhibitor of uPA (5). Binding of uPA to uPAR localizes proteolysis to the cell surface, a process referred to as pericellular proteolysis, while complex formation with inhibitors such as PAI-1 leads to enzyme inactivation (4).

**Figure 1:**
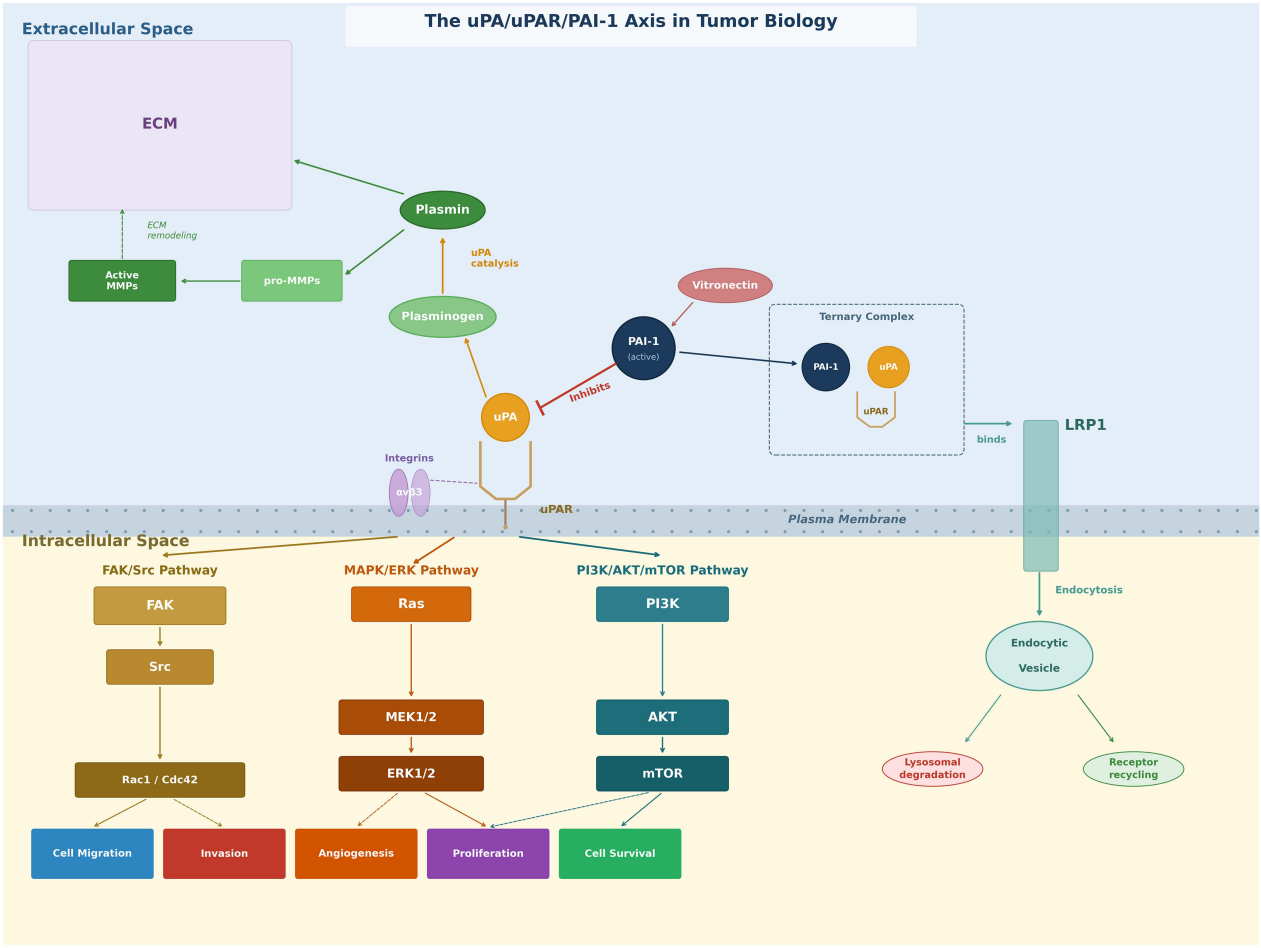
The uPA/uPAR/PAI-1 axis in tumor biology. PAI-1 inhibits plasmin-mediated extracellular matrix (ECM) degradation by blocking plasminogen activation. Despite its antiproteolytic activity, uPAR also initiates intracellular signaling via focal adhesion kinase (FAK), mitogen-activated protein kinase (MAPK), and PI3K/AKT pathways, promoting tumor cell survival, migration, and invasion.

The currently recognized functions of uPA-dependent plasminogen activation are mainly associated with physiological and pathological tissue remodeling processes, including cancer invasion, whereas tissue-type plasminogen activator (tPA) activity is more closely linked to thrombolysis and neurobiology (5). In addition to its proteolytic function, uPAR can also initiate non-proteolytic signaling through interactions with integrins and vitronectin, influencing cell adhesion, migration, and survival (4) (**Figure 1****).**

The plasminogen activation system includes the serpins PAI-1 and PAI-2, both of which belong to the serine protease inhibitor (serpin) family, though each has distinct properties (6, 7). Functionally, PAI-1 efficiently inhibits both uPA and tPA, whereas PAI-2 effectively inhibits uPA but is a weak inhibitor of tPA, highlighting key differences in their biological roles and potential clinical relevance (6, 8).

PAI-1 is the principal physiological inhibitor of uPA and tPA within the plasminogen activation system. Notably, despite this inhibitory role, elevated PAI-1 expression has been repeatedly linked to aggressive tumor behavior and adverse outcomes (9). This apparent contradiction forms the basis of the “PAI-1 paradox,” which is discussed in the following section.

## 3 The PAI-1 paradox: molecular and cellular mechanisms

**Figure 2:**
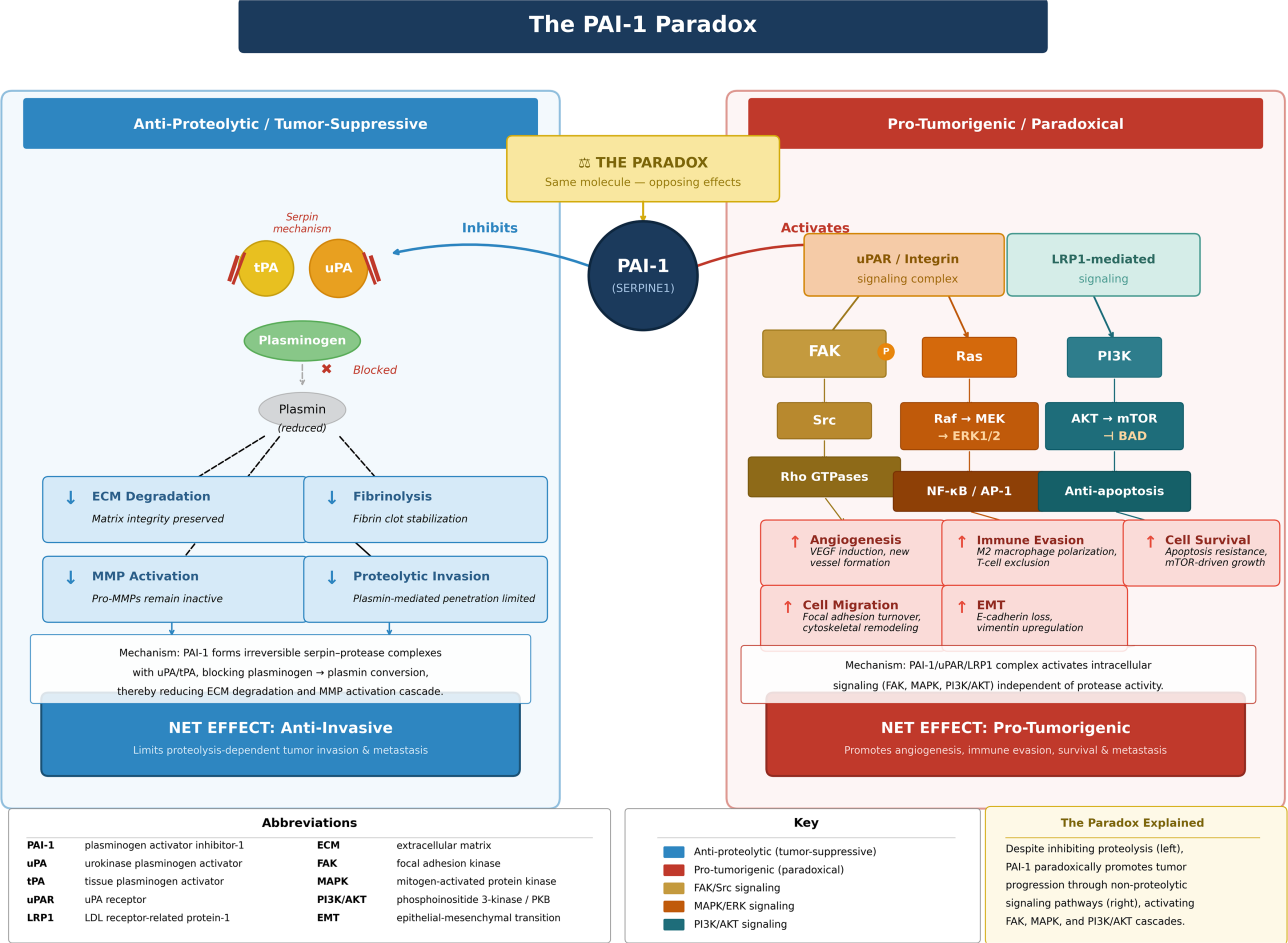
The PAI-1 paradox.PAI-1 inhibits plasmin generation, limiting extracellular matrix (ECM) degradation and proteolysis-dependent invasion. Paradoxically, PAI-1 also promotes pro-tumorigenic signaling through activation of focal adhesion kinase (FAK), mitogen-activated protein kinase (MAPK), and PI3K/AKT pathways, resulting in angiogenesis, immune suppression, and tumor cell survival.

Plasminogen activator inhibitor-1 (PAI-1) inhibits tissue-type (tPA) and urokinase-type (uPA) plasminogen activators, thereby reducing plasmin generation and limiting extracellular matrix degradation. In principle, this should restrain invasion and metastasis. Paradoxically, elevated PAI-1 in tumor tissue and circulation is consistently associated with poor prognosis and therapy resistance across multiple cancers (9) (**Figure 2**). In breast cancer, tumor PAI-1 expression demonstrates independent predictive value in both node-negative (HR = 2.7) and node-positive (HR = 2.4) disease (10), supporting PAI-1 as both a biomarker and a functional mediator of tumor progression. However, across cancer types, much of the mechanistic support for causality comes from preclinical models, and clinical associations often cannot distinguish whether PAI-1 is a driver of aggressiveness or a surrogate of stromal activation and treatment-induced stress.

The urokinase-type plasminogen activator receptor (uPAR) is a key regulator of plasminogen activation, proteolysis, signal transduction, and cell adhesion (11). Classically, uPAR functions as a receptor for the zymogen form of urokinase-type plasminogen activator (pro-uPA), initiating a cascade of proteolytic events that lead to degradation of the extracellular matrix (ECM) (11, 12). Importantly, this uPAR signaling scaffold provides a route by which PAI-1 can exert tumor-promoting effects even while inhibiting proteolysis, helping reconcile the apparent ‘anti-protease’ versus ‘pro-tumor’ contradiction. Through these associations, uPAR activates key intracellular signaling pathways, including focal adhesion kinase (FAK), mitogen-activated protein kinase (MAPK), phosphatidylinositol 3-kinase/protein kinase B (PI3K/AKT), and Janus-associated kinase 1 (JAK1). These pathways drive essential cellular responses such as migration, adhesion, proliferation, angiogenesis, and epithelial–mesenchymal transition (EMT) (11). Finally, uPAR plays a critical role in the regulation and recycling of its own complex. When the uPA-uPAR complex is inhibited by PAI-1, a tripartite uPA-PAI-1-uPAR complex is formed and internalized via the low-density lipoprotein receptor-related protein (LRP) through clathrin-mediated endocytosis. Inside the cell, uPA-PAI-1 dissociates from uPAR and is directed to the lysosome for degradation, while the unoccupied uPAR is efficiently recycled back to the cell surface for future activation cycles (13–15). Notably, the quantitative contribution of this trafficking loop to patient-level outcomes is difficult to infer because uPAR internalization dynamics are highly context-dependent and vary with receptor expression, ligand availability, and microenvironmental composition.

One study revealed that PAI-1 exhibits dose-dependent regulation of angiogenesis. PAI-1 appears to be proangiogenic at physiological concentrations through its antiproteolytic function (16). However, at supraphysiological concentrations, excessive inhibition of uPA and plasmin suppresses the matrix degradation and endothelial invasion required for neovascularization, thus inhibiting angiogenesis (17). Experimental models show this biphasic “bell-shaped” phenomenon, where angiogenesis was stimulated threefold at low or physiological PAI-1 concentrations but was virtually abolished at high concentrations (18). This biphasic behavior also explains why angiogenesis findings can appear inconsistent across studies, since ‘high’ and ‘low’ PAI-1 exposures differ by model system, source of PAI-1 (tumor vs host), and whether measurements capture active versus latent pools.

In addition to concentration-dependent effects, binding interactions also control PAI-1’s cellular effects. Integrin αvβ3 is usually occupied by vitronectin to promote motility. This same site can also be occupied by PAI-1, thereby regulating or inhibiting cell adhesion and migration according to the competitive balance between the two (19, 20). Collectively, these data indicate that PAI-1 effects are conditional rather than uniform: the same molecule can restrict invasion when proteolysis is rate-limiting, yet promote migration and survival when signaling, adhesion turnover, or stromal remodeling dominate.

### 3.1 Adhesion and migration switching

PAI-1 is associated with vitronectin in both plasma and the extracellular matrix, potentially influencing cell-substratum integrity *in vivo*. Recent studies have demonstrated that PAI-1 bound to vitronectin in the ECM can block the binding of integrins and the urokinase plasminogen activator receptor (uPAR) to vitronectin, thereby impeding cell adhesion and migration on vitronectin-coated surfaces. The PAI-1 binding site on vitronectin, located at the edge of β-sheet A, is sensitive to conformational changes in β-sheet A as well as alterations caused by PAI-1 conversion to its latent form or by reactive center loop (RCL) cleavage by a nontarget protease (21).

Evidence also indicates that PAI-1 can promote vitronectin multimerization by forming large higher-order complexes. Moreover, vitronectin–PAI-1 complexes exhibit enhanced binding to GPIIbIIIa in a metal-dependent manner, a novel finding currently being explored through investigations of vitronectin interactions with various integrins under diverse conditions. Paradoxically, tumor cells may exploit this mechanism by engaging PAI-1 and vitronectin interactions, thereby evading the adhesive barriers that typically constrain normal cells and promoting invasiveness (22). This interaction shifts cells from stable adhesion toward a migratory phenotype, providing a mechanistic explanation for how PAI-1 can facilitate invasion despite inhibiting proteolysis.

### 3.2 Angiogenesis regulation

A Matrigel implant assay showed that angiogenesis was approximately three times higher in animals overexpressing PAI-1 and decreased by about 60% in PAI-1–null mice compared with wild-type controls (17). Further supporting this, another study examined the effects of pharmacologically blocking PAI-1 using the inhibitor SK-216 in mice expressing high levels of VEGF and found that SK-216 administration significantly reduced tumor weight and angiogenesis in malignant pleural mesothelioma. In addition, SK-216 inhibited the migration and tube formation of human umbilical vein endothelial cells (HUVECs) in culture when stimulated by angiogenic factors released by malignant pleural mesothelioma cells (23).

### 3.3 Extracellular matrix remodeling

Beyond its effects on adhesion and migration, PAI-1 also influences extracellular matrix architecture. Mechanistically, PAI-1 influences the intracellular activities of β-catenin, which in turn triggers integrin-mediated inside-out signaling to alter the structure of the ECM. Through interactions with the uPA/uPAR plasminogen activator pathway, these effects appear to differ from the classic proteolytic inhibitory activity of PAI-1 (24). Accordingly, this reveals a novel and complex role for PAI-1 in modifying the extracellular environment and regulating its nanomechanical properties, thereby enhancing cellular migration during tumor invasion.

### 3.4 Survival and therapy resistance signaling

By regulating pericellular plasmin activity, PAI-1, similar to endothelial cells, prevents Fas-mediated apoptosis in a variety of human cancer cells, including brain metastases (9, 25). Additionally, intracellular PAI-1 increases cell survival by inhibiting caspase-3, thereby protecting tumor cells from apoptosis induced by chemotherapy (9, 26). PAI-1 also modulates pro-survival signaling pathways. Another study revealed that in MCF-7 breast cancer cells, PAI-1 does not directly activate ERK1/2. Instead, when it forms a complex with uPA, it prolongs ERK activation compared to the transient signal triggered by uPA alone. This sustained signaling requires both the uPA receptor (uPAR) and the very low-density lipoprotein receptor (VLDLr). Blocking VLDLr or preventing PAI-1 binding restores the brief nature of ERK activation (27). Furthermore, the uPA–PAI-1 complex enhances downstream signaling through FAK and Shc, maintaining the activity of the Sos–Shc complex for an extended period and supporting prolonged cell growth and survival signals (27, 28). Beyond tumor-intrinsic signaling, PAI-1 also reshapes the surrounding stroma and immune compartment, reinforcing invasion and therapy resistance.

### 3.5 Tumor microenvironment remodeling

Beyond intrinsic tumor cell signaling, PAI-1 plays a crucial role in the tumor microenvironment, supporting tumor adaptation and survival under stress. Experimental models have demonstrated that tumor growth depends not only on tumor-cell PAI-1 but also on host-derived PAI-1. Loss of PAI-1 in the host microenvironment markedly impairs tumor progression, and tumor-cell production alone cannot compensate for host deficiency. These findings indicate that stromal compartments represent a major functional source of PAI-1 within tumors rather than cancer cells alone (29). Accordingly, measured PAI-1 levels may represent different biological processes depending on the compartment analyzed.

**Figure 3:**
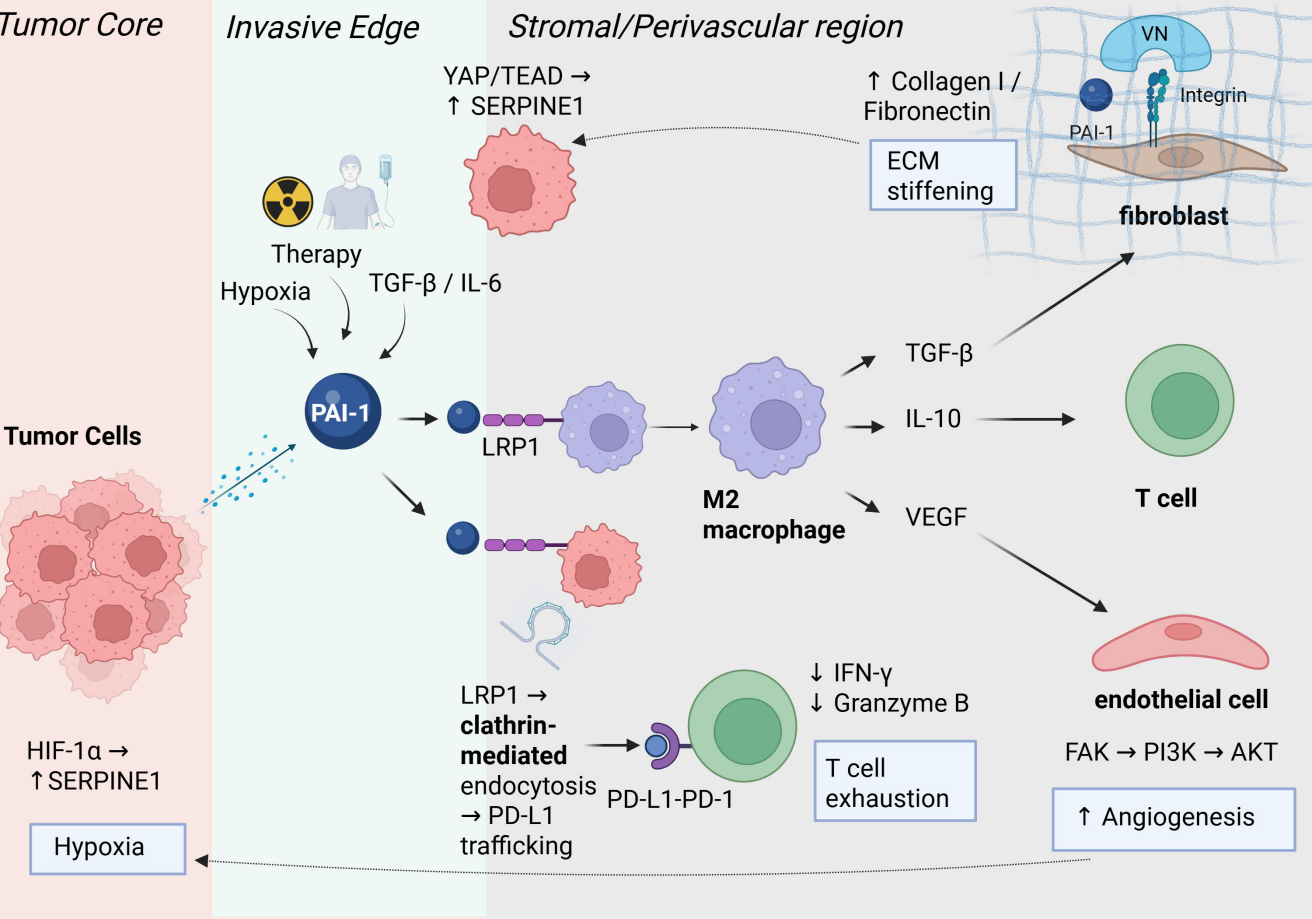
PAI-1 and the tumor microenvironment. Tumor cells secrete PAI-1, which binds to vitronectin (VN) and integrins (αvβ3/β5) on endothelial and fibroblast cells to promote angiogenesis and extracellular matrix remodeling (ECM). PAI-1 also polarizes macrophages towards an M2 phenotype that secretes IL-10, TGF-β, and VEGF, which promote immunosuppression, fibrosis, and angiogenesis, respectively. PAI-1 also promotes immune evasion through the regulation of PD-L1 trafficking through the LRP1 receptor on tumor cells to facilitate PD-L1/PD-1 interaction that inhibits cytotoxic T-cell function. Together, these pathways illustrate how PAI-1 coordinates vascular, stromal, and immune components to create a protumorigenic microenvironment. Created in BioRender.

As a critical regulator of the plasminogen activation cascade, PAI-1 under normal physiological conditions promotes wound healing via extracellular-matrix (ECM) deposition. However, under pathological circumstances, PAI-1 expression over time leads to exuberant ECM deposition, epithelial-to-mesenchymal transition (EMT), and elevated invasiveness, promoting fibrosis and tumor growth (30). PAI-1 can promote attracting and polarizing tumor-associated macrophages (TAMs), which produce pro-angiogenic and pro-inflammatory cytokines such as IL-1, IL-8, and VEGF, thereby supporting the growth of tumors. This pro-tumor activity is further amplified as pro-tumorigenic factors like TGF-β, IL-6, and TNF-α have been shown to stimulate PAI-1 production by endothelial cells, fibroblasts, adipocytes, smooth muscle cells, and macrophages within the tumor microenvironment (9). PAI-1 also triggers intracellular signaling within macrophages, leading to increased immunosuppressive cytokine production, establishing an immune tolerance microenvironment as well as stromal remodeling (31). These converging pathways place PAI-1 as coordinator of tumor-stroma communication, linking fibrinolysis, angiogenesis, and immune regulation within the tumor microenvironment (**Figure 3**).

### 3.6 Immune evasion and checkpoint modulation

PAI-1 is an immunoregulatory molecule that shapes the immune response to tumors. Studies have shown that PAI-1 may function similarly to immune checkpoint molecules by helping cancer and senescent cells to evade immune attack (32). PAI-1 enhances immune evasion by modulating PD-L1 trafficking. Mechanistic studies indicate that PAI-1 induces PD-L1 expression through the JAK/STAT signaling pathway, allowing tumor cells to evade immune surveillance (33).

It was discovered that PAI-1 controls PD-L1 endocytosis. It promotes PD-L1 internalization and lysosomal degradation by clathrin-mediated endocytosis via the LRP1 receptor. This reduces PD-L1 availability on the cell surface. Inhibition of PAI-1 reverses this effect and leads to accumulation of PD-L1 at the plasma membrane and increased sensitivity to anti-PD-L1 checkpoint therapy (34).

Furthermore, tumor-cell-secreted PAI-1 increases PD-L1 expression in cell populations within the tumor microenvironment, including tumor-associated macrophages (TAMs) and cancer-associated fibroblasts (CAFs), allowing tumors to evade immune cell attack (33). In addition, PAI-1 promotes macrophage polarization into an M2 state of immunosuppression, thereby inhibiting cytotoxic T-cell function (34). This evidence suggests that PAI-1 inhibition may prevent PD-L1 induction, have an immunostimulatory effect on diverse cancers, and enhance both anti-senescent and anti-tumor immune responses (33). In combination, these findings redefine PAI-1 not only as a promoter of angiogenesis and metastasis but also as an immune gatekeeper, increasing the therapeutic promise and challenge, of combining PAI-1 blockade with immune checkpoint blockage in cancer.

Taken together, the ‘PAI-1 paradox’ is best viewed as a context problem rather than a true contradiction. Apparent anti-invasive effects tend to emerge in settings where extracellular proteolysis is the dominant bottleneck, whereas pro-tumor associations are more consistently observed when stromal remodeling, survival signaling, immune suppression, or therapy-induced stress responses shape tumor fitness. This framework predicts that clinical translation will require compartment-aware measurement (tumor vs plasma) and form-aware assays (active vs inactive), because total PAI-1 abundance alone may conflate biologically distinct processes.

## 4 PAI-1 in specific tumor contexts

### 4.1 Invasion-dominant epithelial cancers

In epithelial gynecologic and breast cancers, the uPA-uPAR-PAI-1 system is frequently upregulated. In breast cancer, uPAR interacts with HER2 and estrogen receptor (ER) signaling to induce ERK-mediated proliferation and resistance to apoptosis, providing a mechanistic connection between PAI-1 and aggressive behavior. Clinically, elevated uPA and PAI-1 levels independently correlate with adverse relapse-free and metastasis-free survival, independent of nodal status (35). Similarly, in ovarian cancer, PAI-1 correlates with peritoneal metastasis, late stage, and poor survival. Notably, upon PAI-1 silencing, there were significant phenotypical changes. However, platelet addition induced proliferation, suggesting a platelet–PAI-1 cross-talk that can be permissive to metastasis. High plasma PAI-1 was also detected in circulating tumor cells and was reduced after chemotherapy, indicating its potential as a biomarker of tumor load and treatment response (36). PAI-1 and uPAR were found to be overexpressed in cervical and endometrial cancers. This overexpression was correlated with stage, recurrence, and hypoxia-induced invasiveness, implicating a shared function in proteolytic remodeling and epithelial-mesenchymal transition (35). Also, the SERPINE1 rs1799889 (4G/5G) polymorphism, the gene regulating PAI-1 expression, has been associated with enhanced risk of breast and endometrial cancer. The 4G allele increases transcriptional activity and circulating PAI-1 levels. Interactions with TGF-β1 (rs1800468) and uPA (rs4065) variants may further amplify susceptibility, especially in cervical cancer (37). However, the polymorphism associations are modest and population-dependent, indicating that genetic regulation alone does not fully explain PAI-1’s prognostic behavior and reinforcing the importance of microenvironmental regulation.

### 4.2 Therapy-adaptation and hypoxia-associated tumors

In lung cancer, increased expression of PAI-1 is related to advanced stage, adverse prognosis, and resistance to multiple therapies. PAI-1 contributes to radioresistance through hypoxia-induced activation of the AKT/ERK signaling pathway. It promotes chemoresistance by activating cancer-associated fibroblasts (CAFs), which support tumor survival. It also drives targeted therapy resistance by inducing integrin-mediated epithelial–mesenchymal transition (EMT) (38). These findings indicate that in lung cancer, PAI-1 acts predominantly as a treatment-adaptation mediator, linking hypoxia signaling, stromal activation, and therapy resistance rather than serving purely as a prognostic marker.

In glioblastoma multiforme (GBM), PAI-1 overexpression is linked to poor prognosis and mesenchymal transition. Inhibition of PAI-1 suppresses glioma development through the PI3K/AKT pathway and increases immune-stimulatory effect, highlighting its role in tumor progression and immune regulation (39, 40). Furthermore, PAI-1 acts as a stress-response mediator in glioma cells. When autophagy is pharmacologically inhibited, intracellular PAI-1 is upregulated. Importantly, simultaneous inhibition of PAI-1 and autophagy produces a synergistic antitumor effect, promoting a pro-inflammatory immune microenvironment (40, 41). Unlike epithelial cancers, GBM data suggest that PAI-1 is closely linked to mesenchymal transition and stress adaptation rather than metastatic dissemination, indicating that its primary role in brain tumors may be cellular plasticity and therapy tolerance.

### 4.3 Stromal-remodeling and fibrosis-associated tumors

In gastrointestinal tract malignancies, particularly hepatocellular carcinoma and colorectal malignancy, elevated PAI-1 expression has been associated with multifocal disease, invasive tumor phenotype, and reduced survival. In hepatobiliary malignancies, PAI-1 contributes to fibrosis and inflammation, leading to hepatocarcinogenesis; however, its direct oncogenic role remains debated (30). High PAI-1 expression is more frequent in multifocal hepatocellular carcinoma and associates with worse survival (42). Mechanistically, PAI-1 modulates cellular senescence pathways, including YAP-dependent oncogene-induced senescence. This links it to both tumor suppression and progression depending on the biological context (43). In colorectal cancer, elevated PAI-1 expression and 4G promoter polymorphism correlate with invasive phenotype and worse survival, with some effects possibly due to stromal or inflammatory, but not tumor-intrinsic, activity (44). In these tumors, PAI-1 expression frequently reflects fibrotic or inflammatory stromal remodeling rather than tumor cell-intrinsic oncogenic signaling, which may explain why its prognostic strength is weaker and more variable in hepatobiliary malignancies.

### 4.4 Clinical interpretation and biomarker variability

Plasminogen activator inhibitor-1 (PAI-1) overexpression is strongly linked to adverse prognosis in a variety of malignancies, although the strength of association varies based on tumor type and microenvironmental conditions (2). Because PAI-1 is inducible by inflammatory cytokines such as IL-6 and TNF-α, circulating levels may reflect host inflammatory status rather than tumor biology alone, complicating its interpretation as a universal prognostic biomarker. Across tumor types, the prognostic impact of PAI-1 appears inconsistent: in some cancers it correlates strongly with metastasis and therapy resistance, whereas in others it reflects stromal remodeling or inflammation rather than tumor aggressiveness. Importantly, not all clinical datasets demonstrate a uniform adverse prognostic signal for PAI-1. In a cohort of metastatic colorectal cancer patients treated with bevacizumab-containing chemotherapy, baseline plasma PAI-1 levels were associated with treatment response but did not significantly correlate with survival outcomes, indicating limited prognostic value in that setting (45). Similarly, in another clinical biomarker analysis, PAI-1 expression was not associated with overall survival (46).

Taken together across tumor types, the available evidence indicates that PAI-1 does not operate as a universal oncogenic driver across malignancies. Instead, its biological impact depends on the dominant tumor ecology. In epithelial cancers, PAI-1 mainly facilitates invasion and metastatic dissemination by promoting adhesion turnover and survival signaling (37). In contrast, in hypoxic and treatment-stressed tumors such as lung cancer and glioblastoma, PAI-1 functions primarily as a mediator of therapy adaptation and resistance (38, 41). In hepatobiliary and colorectal tumors, however, PAI-1 expression more often reflects stromal fibrosis and inflammatory remodeling rather than tumor-intrinsic oncogenic signaling (30). These context-dependent roles help explain why PAI-1 serves as a strong prognostic biomarker in certain cancers but shows weaker or inconsistent predictive value in others. Accordingly, interpretation of PAI-1 levels should be tumor-specific and mechanism-guided rather than generalized across malignancies (**Table 1**).

**Table 1:** Tumor-specific PAI-1 (SERPINE1) expression, biological mechanisms, clinical associations, and level of evidence.

Cancer type	Mechanism	Clinical correlation	Level of evidence	References
Breast Cancer	uPA-uPAR-PAI-1 system → ERK-mediated proliferation and resistance to apoptosis	Higher PAI-1 associates with worse relapse-free survival and overall survival; particularly in node–negative disease	Retrospective clinical	(79)
Ovarian Cancer	platelet–PAI-1 crosstalk that enhances migration/EMT and metastasis	High PAI-1 correlates with advanced stage, peritoneal spread, and poorer survival; decreases after neoadjuvant chemotherapy, suggesting it is a biomarker of tumor load	Clinical observational + translational	(36)
Endometrial and Cervical Cancer	uPA–uPAR–PAI-1 axis upregulated under hypoxia/inflammation drive→ invasion and ECM remodeling	Overexpression correlates with higher stage and recurrence risk; evidence heterogeneous by tumor type	Retrospective clinical	(35) (37)
Colorectal Cancer	PAI-1 overexpression promotes tumor invasion and metastasis via uPA-uPAR-mediated ECM degradation and angiogenesis	Higher SERPINE1/PAI-1 expression is associated with more advanced stage/metastasis and poorer survival	Retrospective clinical	(37) (44)
Glioblastoma Multiforme (GBM)	PAI-1 promotes invasion, proliferation, and migration via activation of the PI3K/AKT signaling pathway, and recruits mast cells through LRP1-mediated STAT3 activation	High SERPINE1/PAI-1 expression predicts worse overall survival; PAI-1 inhibition reduces proliferation/invasion in preclinical models	Preclinical + translational	(39) (40) (41)
Lung Cancer	PAI-1 → angiogenesis, EMT, and therapy resistance: – ↑ Radioresistance (hypoxia → AKT/ERK) – ↑ Chemoresistance (via CAF activation) – ↑ Targeted therapy resistance (integrin-mediated EMT)	Higher PAI-1 expression commonly associated with advanced stage and poor prognosis (review)	Preclinical + translational	(38)
Hepatobiliary Cancer	PAI-1 involved in fibrosis–inflammation crosstalk; YAP/TEAD-driven SERPINE1 transcription; ECM deposition	Higher PAI-1 expression often linked to multifocal disease and poorer survival	Preclinical + retrospective clinical	(30) (42) (43)

PAI-1, plasminogen activator inhibitor-1; SERPINE1, serpin family E member 1; uPA, urokinase-type plasminogen activator; uPAR, urokinase plasminogen activator receptor; ECM, extracellular matrix; EMT, epithelial–mesenchymal transition; ERK, extracellular signal-regulated kinase; PI3K, phosphoinositide 3-kinase; AKT, protein kinase B; LRP1, low-density lipoprotein receptor-related protein 1; STAT3, signal transducer and activator of transcription 3; CAF, cancer-associated fibroblast; YAP, Yes-associated protein; TEAD, TEA domain transcription factor; GBM, glioblastoma multiforme.

## 5 Therapeutic strategies targeting PAI-1

PAI-1 has become an attractive therapeutic target. To block the actions of PAI-1, multiple therapeutic approaches have been developed, particularly small molecules that directly inhibit PAI-1 (47).

### 5.1 Small-molecule inhibitors

Tiplaxtinin (PAI-039) is an indole oxoacetic acid derivative and a small molecule that is designed to selectively inhibit plasminogen activator inhibitor-1 (PAI-1), the primary protein that prevents fibrinolysis. Tiplaxtinin binds tightly to the active form of PAI-1 but binds weakly to the inactive (latent) form. It also demonstrates a high level of specificity, meaning that it had no effect against more than 40 other proteins.

In preclinical animal studies, oral administration improved blood flow and inhibited thrombus formation without increasing blood pressure or bleeding risk. After oral administration, the drug was rapidly absorbed through the gastrointestinal tract, and a plasma half-life of 3–5 hours was observed in both rats and dogs. Toxicology testing revealed a wide safety margin; even doses hundreds of times higher than therapeutic levels caused no cardiovascular, neurologic, or systemic toxicity. These findings together show that Tiplaxtinin is well absorbed, highly selective, and safe, which supports its potential for further clinical development (48). However, these studies also indicated that sustained tumor suppression would require inhibitors with improved pharmacokinetic stability and antitumor efficacy.

Other compounds that have been investigated are TM5275 and TM5441. TM5275 and TM5441 are orally active small-molecule inhibitors designed to block plasminogen activator inhibitor-1 (PAI-1). Compared with the Tiplaxtinin, they show better pharmacokinetic behavior and stronger selectivity for the active form of PAI-1. Both agents successfully reduce PAI-1 activity and have demonstrated kidney protective and anti-fibrotic effects in diabetic mouse models, without any evidence of increased bleeding risk. In pharmacokinetic studies, TM5441 achieved its maximum plasma concentration approximately one hour after oral administration, followed by a significant decrease over the subsequent day, indicating relatively rapid systemic clearance (49). In preclinical models, small molecules promoted cancer cell death and disrupted tumor blood vessels, leading to slower tumor growth. Although overall tumor shrinkage was limited, due to the drug staying in the body for a short time, TM5441 showed clear inhibition of tumor progression in HT1080 (fibrosarcoma) and HCT116 (colorectal cancer) models. These results suggest that blocking PAI-1 can slow cancer growth by affecting cell survival, division, and blood vessel formation, making it a promising supportive cancer therapy (50). Furthermore, studies in ovarian cancer cells showed that blocking PAI-1 pharmacologically produced effects comparable to genetic PAI-1 deficiency, resulting in reduced proliferation (51). Blocking PAI-1 with TM5441 greatly reduced tumor size and metastasis in mouse models of peritoneal cancer, even when standard chemotherapy did not work well (52). However, small-molecule inhibition alone may not fully suppress extracellular or stromal PAI-1 activity within tumors.

### 5.2 Biologic and targeted degradation approaches

**Figure 4:**
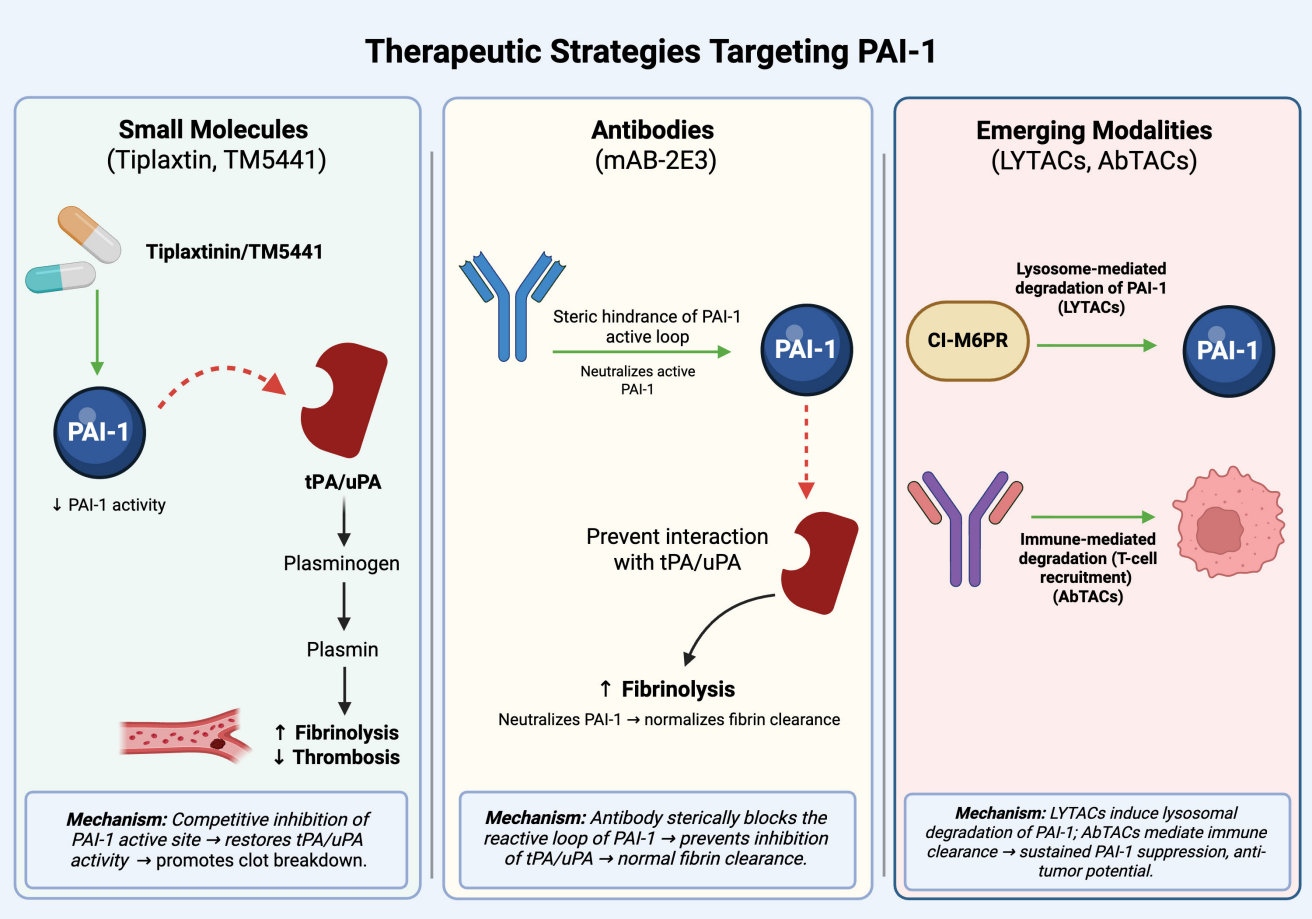
Therapeutic strategies targeting PAI-1. PAI-1 can be inhibited by multiple approaches, including small-molecule inhibitors that block protease interactions, monoclonal antibodies that neutralize active PAI-1, and targeted degradation strategies such as lysosome-targeting chimeras (LYTACs) and antibody-targeted chimeras (AbTACs). These strategies aim to restore fibrinolysis and reduce PAI-1–mediated tumor-promoting signaling. Created in BioRender.

Beyond small-molecule inhibitors, additional biologic and targeted degradation approaches have been developed to more selectively suppress extracellular PAI-1 activity. Additional preclinical studies have demonstrated that an antibody mAb-2E3 targets the active form of PAI-1 and prevents it from binding to Low-density lipoprotein receptor-related protein 1 (LRP1), which limits invasion and lung metastasis in esophageal cancer models (53). Researchers looked into novel methods to selectively break down PAI-1 using techniques like lysosome-targeting chimeras (LYTACs) and antibody-targeted chimeras (AbTACs) (**Figure 4****).** This facilitates the protein being endocytosed by the cell and sent to the lysosome for degradation. This leads to an effective decrease of extracellular PAI-1 levels, which demonstrates the viability of targeted protein degradation for oncological purposes. While these degraders have revealed encouraging results in early studies, achieving effective delivery of these agents to their extracellular targets remains a significant challenge. More research is required to improve their design, selectivity, and develop methods to facilitate intracellular uptake. Together, these approaches indicate that therapeutic targeting of PAI-1 is evolving from pharmacologic inhibition toward precision extracellular protein modulation (**Table 2**).

**Table 2:** – PAI-1 inhibitors and pharmacologic profiles.

Strategy	Compound	Target/mechanism	Developmental stage	Study design	Sample size	Primary endpoints	Key effects	Critical evaluation	References
Small-molecule PAI-1 inhibition	Tiplaxtinin (PAI-039)	Selective active-site PAI-1 inhibitor; binds active conformation; induces substrate behavior and prevents stable PAI-1–protease complex formation; binding mutually exclusive with vitronectin	Preclinical	*In vitro* biochemical assays + *in vivo* acute arterial thrombosis models (rat carotid injury; canine coronary injury)	Rats: n=4/group; Dogs: n=3/group	*In vitro*: IC_50_, Kd; *In vivo*: time to occlusion, arterial flow reduction, thrombus weight, reperfusion	IC_50_: 2.7 µM; Kd: 480 nM (active PAI-1); ↑ occlusion time (rat: 50.5 ± 9.5 vs 11.5 ± 0.4 min control); ↓ thrombus weight; spontaneous reperfusion in dogs (3/3 treated vs 0/3 controls); no bleeding or BP changes reported	Evidence limited to small preclinical cohorts; efficacy demonstrated only in acute thrombosis models; no chronic disease or survival data; micromolar potency; reversible binding may require sustained exposure; absence of human clinical validation	(48) (80)
Small-molecule PAI-1 inhibition	TM5275	Orally bioavailable small-molecule PAI-1 inhibitor; blocks PAI-1 activity and downstream profibrotic and pro-angiogenic signaling	Preclinical	*In vitro* cancer cell assays; endothelial branching assays; HT1080 & HCT116 xenografts (mouse); CDAA and PS-induced fibrotic rat models	Xenografts: n=6/group; Fibrosis models: n=6/group	IC_50_ (tPA-dependent assay); cell viability; caspase activation; CD31 density; Sirius Red fibrosis area; Tgfb1/Col1a1 expression	IC_50_ = 6.95 µM (tPA-dependent hydrolysis); ↓ tumor cell viability (IC_50_ range 9.7–60.3 µM depending on cell line); ↓ endothelial branching; ↓ vessel density; trend toward ↓ tumor growth (not statistically significant); ↓ liver fibrosis (~50% reduction in fibrotic area); ↓ TGF-β1 and collagen expression	Antitumor activity modest and not statistically significant *in vivo*; requires high micromolar concentrations *in vitro*; plasma trough levels undetectable; effects primarily preclinical; stronger antifibrotic than antitumor signal; no human clinical data	(50) (81)
Small-molecule PAI-1 inhibition	TM5441	Orally bioavailable PAI-1 inhibitor; blocks PAI-1 activity; disrupts pro-angiogenic and STAT3-associated signaling; inhibits endothelial branching and tumor-supportive paracrine signaling	Preclinical	*In vitro* cancer cell assays; endothelial Matrigel assays; HT1080 & HCT116 xenograft mouse models; patient-derived ascites-dependent peritoneal carcinomatosis models	Xenografts: n≈6/group; PC models: multiple patient-derived ascites samples; *in vitro* quadruplicate assays		IC_50_: 9.7–60.3 µM; ↑ apoptosis (50 µM); ↓ endothelial branching; ↓ CD31+ vessel density; ↓ tumor growth (not statistically significant); no increased bleeding; peak plasma ~11.4 µM, undetectable at 23 h; selective efficacy in PAI-1–high ascites models	Antitumor efficacy modest and not statistically significant in xenografts; requires high µM concentrations *in vitro*; short systemic exposure; efficacy context-dependent (PAI-1–high tumors); preclinical only; no human clinical validation	(50) (81) (52)
Monoclonal antibody therapy	mAb-2E3	Monoclonal antibody against active PAI-1; blocks interaction with LRP1 and downstream STAT1 signaling	Preclinical	*In vitro* migration and invasion assays; wound healing; tail-vein metastasis model; subcutaneous tumor model in SCID/Beige mice	*In vivo*: ~n ≥ 6–8 per group (40 mg/kg vs control; lung metastasis and tumor growth models)	Inhibition of metastasis (lung IVIS signal); tumor volume and weight reduction; cell migration/invasion	↓ tumor growth (~35% inhibition at 40 mg/kg); ↓ lung metastasis burden; ↓ migration and invasion in ESCC cells; ↓ STAT1 phosphorylation; blocks PAI-1–LRP1 binding	Preclinical only; antitumor signal dose-dependent; no human data; exact KD/IC_50_ for antibody not reported; mechanistic outcome partly surrogate (binding/STAT1)	(53)
Targeted protein degradation	LYTACs (PAI-1–directed lysosome-targeting chimeras)	Bifunctional chimera: one end binds extracellular PAI-1; other recruits CI-M6PR to mediate endocytosis and lysosomal degradation	Early preclinical	Cell-based degradation assays; *in vitro* extracellular protein depletion models	*In vitro* replicates; no animal models reported	Reduction of extracellular PAI-1 levels; receptor-mediated internalization	↓ extracellular PAI-1 via CI-M6PR-dependent lysosomal degradation; proof-of-concept for targeting secreted PAI-1	Very early stage; no *in vivo* cancer efficacy data; affinity metrics for PAI-1 binding not reported; delivery and tissue penetration remain major challenges; potential off-target effects due to CI-M6PR ubiquity; translational feasibility untested	(82) (83)
Targeted extracellular protein degradation	AbTACs (antibody-targeted chimeras)	Bispecific antibody platform linking extracellular PAI-1 to membrane E3 ligase/receptor to induce internalization and degradation	Early preclinical	*In vitro* proof-of-concept degradation assays	*In vitro* replicates; no animal models reported	Reduction of extracellular target protein levels; receptor-mediated degradation	↓ extracellular PAI-1 levels (proof-of-concept); selective target engagement; potential for extracellular protein removal	Very early-stage technology; no *in vivo* cancer efficacy data; no reported PAI-1 binding affinity metrics; delivery and tissue penetration challenges; no clinical validation	(83)

AbTAC, Antibody-targeted chimera; BP, Blood pressure; CD31, Cluster of differentiation 31 (platelet endothelial cell adhesion molecule-1); CI-M6PR, Cation-independent mannose-6-phosphate receptor; ESCC, Esophageal squamous cell carcinoma; IC_50_, Half maximal inhibitory concentration;IVIS, *In vivo* imaging system; Kd, Dissociation constant; LRP1, Low-density lipoprotein receptor-related protein 1; LYTAC, Lysosome-targeting chimera; PAI-1, Plasminogen activator inhibitor-1; PC, Peritoneal carcinomatosis; STAT1, Signal transducer and activator of transcription; STAT3, Signal transducer and activator of transcription 3; TGF-β1, Transforming growth factor beta 1; tPA, Tissue plasminogen activator.

## 6 Safety and pharmacologic considerations

PAI-1 is an important regulator of fibrinolysis. Elevated PAI-1 levels contribute to a prothrombotic state by inhibiting fibrinolysis, promoting thrombus formation, and accelerating atherosclerotic plaque development. This consequently increases the risk of cardiovascular events, including coronary heart disease and stroke (54). These physiologic functions create important constraints when attempting systemic pharmacologic inhibition. Although the therapeutic rationale for PAI-1 targeting is compelling, translation remains pharmacologically challenging. Current inhibitors have a short *in vivo* half-life (2–3 hours) and are active only at micromolar levels, limiting their therapeutic potential. Moreover, PAI-1’s vitronectin-bound form, which is common in solid tumors, is refractory to inhibition, diminishing drug effectiveness within the ECM-rich tumor microenvironment. Finally, because PAI-1 is an essential regulator of fibrinolysis, sustained systemic inhibition is of theoretical concern for spontaneous bleeding, subject to careful safety evaluation (9).

### 6.1 Measurement and assay considerations

PAI-1 exists in three conformational forms: active, latent, and cleaved. At 37 °C, the active form of PAI-1 has a half-life of approximately 1–2 hours before spontaneously converting into the latent form (21). The latent conformation may play a role in regulating PAI-1 activity. Latent PAI-1 can partially revert to its active state following denaturant treatment (55).

Cleaved PAI-1, on the other hand, is generated either by slow deacylation of the enzyme-inhibitor complex or through reactions with non-target proteases such as elastase, which cleaves the reactive center loop (RCL) at a site distinct from the P1–P1′ bond (56). Both the latent and cleaved conformations are inactive since their RCLs are fully inserted into β-sheet A, rendering them unable to react with proteinases. In contrast, the RCL of active PAI-1 remains exposed, allowing efficient interaction with target proteases (57).

Quantifying active PAI-1 remains challenging. A novel ELISA based on a highly specific capture agent capable of binding active PAI-1 with high affinity was developed to improve detection accuracy. In this study, a stable PAI-1 mutant (PAI-1-disu) was used as a calibrator to enhance assay reproducibility. Using this approach, researchers found that prolonged plasma storage led to a significant reduction in measurable active PAI-1 levels, underscoring the importance of standardized handling conditions for clinical investigations. Sample handling critically affects PAI-1 quantification. It has been shown that plasma PAI-1 levels reduce by approximately 50% after six hours of storage at room temperature. Repeated freeze-thaw cycles of samples or assay calibrators also result in significant loss of activity and should be strictly avoided (58).

Additionally, PAI-1 expression follows a circadian rhythm, with peak levels in the morning that are independent of the sleep-wake cycle. The circadian system, along with factors such as age and activity level, contributes to daily variations in PAI-1. These findings suggest that standardization of the time when drawing blood for PAI-1 measurement is of high importance (59, 60). Finally, methodological interpretation must consider that plasma and platelet PAI-1 represent two distinct biological pools. Studies investigating the relationship between circulating PAI-1 and thrombotic disease should account for contributions from both compartments.

## 7 Clinical landscape, translational gaps, and future directions

### 7.1 Clinical evidence in humans

The clinical development of PAI-1 inhibitors represents a growing area of interest in oncology. PAI-1 plays a central role in pathways that drive tumor invasion, blood-vessel growth, and metastatic spread, making it an appealing target for cancer therapy. However, turning these biological findings into clear clinical benefit has proven difficult (61).A key advancement toward translating PAI-1 inhibition into clinical oncology was the initiation of a phase II trial investigating TM5614, a small-molecule PAI-1 inhibitor, in combination with nivolumab for patients with advanced non-small cell lung cancer (NSCLC) who had already received standard chemotherapy and immune-checkpoint blockade. TM5614 showed a favorable safety profile in earlier human studies and improved the effectiveness of PD-1 blockade in preclinical lung cancer models by reshaping the tumor immune microenvironment. In this clinical study, patients received nivolumab alongside oral TM5614 (120 mg daily, increased to 180 mg when tolerated). The main goal was to assess treatment response, while progression-free survival, overall survival, and safety were secondary measures. This was the first clinical study to explore PAI-1 inhibition alongside immunotherapy in NSCLC, with the goal of addressing immune resistance by altering the tumor microenvironment (62).

In addition, a similar phase II multicenter study in Japan examined TM5614 in combination with nivolumab in patients with unresectable melanoma who no longer responded to anti–PD-1 therapy. In that study, treatment responses were observed in approximately one quarter of patients, and disease control was achieved in about two-thirds, with median progression-free and overall survival durations of 5.8 and 9 months, respectively. Most adverse events were mild, and no participants discontinued therapy due to TM5614. These findings suggest that PAI-1 inhibition may help restore immune responsiveness in advanced melanoma, warranting the need for validation in larger controlled trials (63).

Before being tested in oncology, TM5614 was tested in humans for non-cancer conditions, providing essential safety data. A randomized, double-blind, placebo-controlled phase II trial enrolled 75 patients with mild to moderate COVID-19 pneumonia who received oral TM5614 (120–180 mg daily for 14 days). The treatment was well tolerated, with no serious adverse effects occurring. Although the study did not show a statistically significant difference in clinical outcomes between groups, it is confirmed that TM5614 can be safely administered in humans at therapeutic doses (64). In summary, these trials show that TM5614 is moving from experimental validation to early human testing. Current clinical experience with PAI-1 inhibition remains limited to small, open-label studies. The TM5614-nivolumab trials in melanoma and NSCLC confirmed safety but were underpowered to define efficacy. Advancement will rely on more extensive, biomarker-driven phase III trials to determine clinical efficacy and enhance patient-selection strategies. Accordingly, defining reliable biomarkers for patient selection represents the next critical step in clinical translation.

### 7.2 Biomarkers and measurement challenges

Despite encouraging results, several factors still limit the clinical success of PAI-1 inhibitors. While initially expected to suppress tumor growth by blocking the uPA pathway, higher levels of PAI-1 have consistently been linked to more aggressive disease behavior and reduced sensitivity to chemotherapy. The paradoxical behavior of PAI-1 depends on its concentration, cellular origin, and tumor microenvironment context. Its interactions with partners such as vitronectin, uPAR, and LRP1 influence cell adhesion, migration, and survival, which vary across tumor types (2). Another key gap is the lack of reliable biomarkers that can identify which patients are most likely to benefit. Preliminary investigations have examined plasma PAI-1 concentrations and immune-related cytokines, but these remain exploratory (62, 65).

The functional inhibitory ability of the PAI-1 molecule is represented by PAI-1 activity, which also reflects antigen concentration; however, PAI-1 antigen concentration levels cannot distinguish between the PAI-1 protein forms (inert, active, latent, and substrate noninhibitory). Therefore, in biological systems and *in vivo* investigations, it is more relevant to investigate PAI-1 activity rather than PAI-1 antigen concentration (66).

Identifying fit-for-purpose pharmacodynamic (PD) markers is important for translating molecular insights into measurable clinical outcomes and for optimizing patient selection and therapeutic monitoring. In support of this, a recent phase I clinical trial investigating the novel drug VT1021 in glioblastoma (GBM) utilized PAI-1 as a predictive biomarker, in which reduced baseline PAI-1 plasma levels were associated with improved clinical response to VT1021 (67). Together, these findings support the role of PAI-1 as a predictive biomarker of therapeutic response. Furthermore, in combination with other circulating tumor indicators, plasma PAI-1 activity has also been evaluated as a hemostatic biomarker in early non-small cell lung cancer (NSCLC) (66). To date, *in vivo* clinical investigations have examined both the tissue expression of PAI-1 and the blood levels of PAI-1 antigen in NSCLC, with generally good correlations (68, 69). Beyond these findings, PAI-1 expression has been linked to therapeutic responsiveness to anti–PD-1 antibodies in advanced melanoma. Current data indicated a substantial correlation between the effectiveness of anti-PD1 Abs in patients with advanced melanoma and the expression levels of PAI-1 on melanoma cells and circulating PAI-1 levels (31).

When selecting patients, it is essential to consider additional prognostic factors that may influence PAI-1 levels and the associated molecular pathways. One study was investigating PAI-1 as a biomarker for resectable non-small cell lung cancer applied exclusion criteria to patients with cardiovascular disease (CVD), prior malignancy, diabetes mellitus (DM), endocrine disorders, hypertension, autoimmune, renal and hepatic disorders, hematologic (including coagulation disorders), neurologic, muscular or psychiatric disorders, HIV infection, recent infection at the time of blood drawn, asthma and alcoholism; if they routinely received any drugs including drugs for hyperlipidemia; and if they were pregnant and in postpartum period for women (66). These findings emphasize that comorbid conditions significantly influence circulating PAI-1 levels and must be considered when using PAI-1 as a clinical biomarker. Accordingly, integrating biomarker-guided patient selection with therapeutic strategies will be essential for maximizing the clinical impact of PAI-1 inhibition.

Clinically, biomarker interpretation should consider both the source and functional state of PAI-1. Tumor-tissue expression most closely reflects tumor invasion biology, whereas circulating plasma PAI-1 often reflects host inflammatory or stromal responses. Measurement of active PAI-1 may therefore be more informative than total antigen, particularly during therapy, since anticancer treatments frequently induce PAI-1 as part of adaptive resistance. Serial assessment before and during treatment could help distinguish aggressive baseline tumor biology from therapy-induced resistance and guide the use of combination strategies incorporating PAI-1 inhibition.

### 7.3 Spatial and nuclear biology of PAI-1

Beyond regulating proteolysis, emerging evidence indicates that PAI-1 exerts compartment-specific functions across the extracellular matrix, cell surface, and nucleus. These spatially distinct roles suggest that PAI-1 organizes tumor behavior not only through enzymatic inhibition but also through structural and transcriptional regulation.

PAI-1 binding to fibrin clots is mediated by vitronectin. Although there are no imaging probes that specifically target PAI-1, a number of studies have shown its downstream matrix components, including fibrin and fibronectin, which are regulated by PAI-1 activity. CLT1 and CLT2 peptides bind with high affinity for fibronectin-fibrin complexes, which are abundant in tumor stroma and show minimal nonspecific binding to normal tissues. By conjugating these peptides to Gd (III) chelates, researchers developed targeted MRI contrast agents capable of selectively enhancing tumor visualization, thereby improving the specificity of tumor imaging. Tumor angiogenesis may also be described by MR molecular imaging of fibrin-fibronectin complexes in tumor tissue using CLT1-(Gd-DTPA) (70).

In addition, a novel study discussed that, to detect orthotopic and metastatic breast cancer and to guide surgical resection using fluorescence images, a new fibronectin-targeted and MMP-9-activatable imaging probe called CREKA-GK8-QC was created. With the help of ex vivo image analysis and *in vivo* preclinical 4T1 orthotopic and metastatic tumor mice models, CREKA-GK8-QC showed significant promise for intraoperative image-guided breast cancer surgery. Collectively, these findings suggest that this dual-targeted fluorescence imaging probe can precisely target fibronectin and MMP-9, allowing for the very specific intraoperative diagnosis and surgery of breast cancer (71).

Although these probes do not directly target PAI-1, they visualize fibrin–fibronectin matrices generated downstream of PAI-1 activity, effectively providing a functional readout of PAI-1-mediated stromal remodeling. Given that PAI-1 regulates the molecular interactions related to fibrin and fibronectin, these imaging techniques may also be used to evaluate PAI-1-associated changes in tumor fibrin architecture.

Emerging evidence revealed that PAI-1 suppresses the expression of genes by acting as a silencer. Accordingly, nuclear PAI-1 overexpression was associated with repression of multiple gene programs (72).These findings indicate that PAI-1 may function as a transcriptional regulator, linking extracellular matrix signaling to direct control of tumor gene expression programs. Together, these observations support a model in which PAI-1 functions as a multi-compartment signaling regulator, coordinating extracellular matrix organization, cellular behavior, and nuclear transcriptional responses.

### 7.4 Therapeutic strategies and combinations

Anticancer therapies consistently activate cellular stress responses within tumors. A common consequence of these responses is induction of PAI-1, which promotes tumor survival, stromal protection, and therapy resistance (73). Therefore, PAI-1 inhibition represents a strategy not as a stand-alone therapy but as a treatment sensitizer that enhances the efficacy of existing anticancer modalities.

This adaptive resistance mechanism is particularly evident in immunotherapy. Checkpoint inhibitors, such as anti–PD-1 or anti– PD-L1 antibodies, block the interaction between PD-1 and PD-L1, resulting in the reactivation of T cells (33, 74). Meanwhile, PAI-1 inhibition remodels the fibrotic and hypoxic tumor microenvironment, thereby facilitating T-cell infiltration (33). Supporting this concept, a study reported that elevated PAI-1 levels correlate with resistance to anti-PD-1 antibody treatment in lung cancer cells. Consistently, an *in vivo* study using a mouse model revealed that the combination of TM5614, a PAI-1 inhibitor, and an anti-PD-1 antibody resulted in greater antitumor efficacy compared to treatment with the anti-PD-1 antibody alone (62).

A similar pattern is observed in angiogenesis-targeted therapy. The PAI-1 inhibitor SK-216 was shown in malignant pleural mesothelioma (MPM) models to inhibit angiogenesis and markedly suppress tumor progression *in vivo*, demonstrated by reduced endothelial migration and tube formation in HUVEC assays. In contrast to bevacizumab, which was effective only in VEGF-dominant tumors, SK-216 inhibited angiogenesis irrespective of the angiogenic factor (VEGF, bFGF, PDGF-BB, or HGF), indicating that PAI-1 inhibition targets a common downstream step in the angiogenic cascade and may offer broader anti-angiogenic potential than a VEGF-specific agent (23). This provides a mechanistic rationale for combining PAI-1 inhibitors with anti-VEGF agents.

Cytotoxic chemotherapy further illustrates this PAI-1-mediated adaptive feedback. PAI-1 inhibitors can be integrated into chemotherapy regimens to enhance efficacy and reduce resistance by targeting several tumor-promoting pathways such as survival signaling, oxidative stress suppression, and extracellular matrix remodeling. Preclinical studies have shown beneficial interactions between PAI-1 inhibitors and multiple chemotherapeutic classes, supporting its translational potential in combination therapy approaches (75).

Among platinum-based chemotherapeutics, both cisplatin and oxaliplatin demonstrate evidence of synergy with PAI-1 inhibition across several tumor models. In esophageal squamous cell carcinoma (ESCC), cisplatin-activated cancer-associated fibroblasts (CAFs) secrete PAI-1 following DNA-damage-induced p53/p21 activation. This extracellular PAI-1 activates the AKT/ERK1/2 pathway and suppresses caspase-3 and ROS, leading to reduced DNA damage and apoptosis in neighboring tumor cells. The PAI-1 inhibitor, tiplaxtinin, restored cisplatin sensitivity *in vitro*, *in vivo*, and in patient samples by increasing ROS levels and apoptosis and decreasing tumor growth (76). Collectively, these studies indicate that DNA-damage therapies induce stromal PAI-1 secretion, which activates pro-survival signaling and suppresses apoptosis, thereby creating a reversible chemotherapy-resistant state. Concurrent administration of PAI-1 inhibitors with cytotoxic drugs, rather than sequential dosing, appears most rational to block this adaptive feedback. Similar sensitizing effects of PAI-1 inhibition have been observed across multiple additional tumor models, supporting that therapy-induced PAI-1 represents a generalizable mechanism of chemotherapy resistance rather than a tumor-specific phenomenon.

Radiotherapy represents another context in which therapy-induced PAI-1 promotes tumor persistence. Radiation induces secretion of PAI-1 from radioresistant NSCLC cells, which affects neighboring radiosensitive NSCLC cells in a paracrine manner to enhance survival and EMT. This effect is mediated by PAI-1 via uPAR/LRP-1 receptor complexes, activating the AKT/ERK1/2 and Snail pathways that enhance cell motility. Irradiated cells exposed to PAI-1-containing conditioned medium exhibited increased migration and mesenchymal marker expression, effects that were reversed by the PAI-1 inhibitor tiplaxtinin. This demonstrates that PAI-1 secretion drives therapy resistance via the PAI-1/AKT/ERK/Snail axis. According to preclinical studies, combining radiotherapy with tiplaxtinin can enhance radiosensitivity and apoptosis (77). Concurrent administration of PAI-1 inhibitors during radiotherapy appears rational to block radiation-induced PAI-1 signaling, although no optimized schedule is established yet (38). Although similar radiation-induced PAI-1 upregulation has been observed in other cancers, such as head and neck SCC, direct combination studies with PAI-1 inhibitors remain lacking (78). Thus, radiation not only kills tumor cells but also paradoxically induces a protective paracrine PAI-1 signaling loop that promotes epithelial–mesenchymal transition and survival in neighboring cells.

Across therapeutic modalities, a consistent pattern emerges: anticancer treatments induce PAI-1 expression, and the resulting signaling protects tumors through stromal remodeling, immune suppression, and survival pathway activation. Consequently, PAI-1 functions as a shared resistance mediator rather than a pathway specific to a single treatment type. Targeting PAI-1, therefore, offers a unifying strategy to sensitize tumors to immunotherapy, chemotherapy, anti-angiogenic therapy, and radiotherapy (**Table 3**).

**Table 3:** Overview of combination strategies involving PAI-1 inhibition across tumor models.

Combination	Agents	Model	Proposed mechanism and key findings	Translational status	Reported combination effect	References
PAI-1 inhibitor + anti-PD-1	TM5614 + anti-PD-1 Ab	MC38 (ICI-sensitive) and B16F10 (ICI-resistant) murine models	Tumor regression; complete disappearance in combination group	*In vivo*	*In-vivo* synergy demonstrated	(33)
PAI-1 inhibitor + anti-VEGF (bevacizumab)	SK-216 + bevacizumab	MPM and HUVEC models	PAI-1 inhibition blocks a common downstream step in angiogenesis independent of VEGF, complementing anti-VEGF agents by broadening angiogenic pathway suppression, SK-216 reduces angiogenesis and tumor growth as monotherapy; no direct in-vivo combination data	*In vivo* (monotherapy); mechanistic rationale for combination	Complementary anti-angiogenic rationale; no direct *in-vivo* combination data reported	(23)
PAI-1 inhibitor + chemotherapy (cisplatin)	Tiplaxtinin + cisplatin	ESCC models	Inhibits CAF-secreted PAI-1/AKT/ERK1/2 pathway and its oxidative stress suppression, reversing CAF-mediated resistance by ↑ROS, ↑ apoptosis, ↓ tumor growth	*In vitro*/*In vivo*	Synergy demonstrated; ↓ tumor growth	(76)
	ACT001 + cisplatin	U118 glioma model	ACT001 inhibits PI3K/AKT pathway, ↓ tumor volume and weight, ↓ angiogenesis, migration and proliferation	*In vivo*	Clear *in-vivo* synergy; potential to overcome platinum resistance	(39)
	SK-216 + cisplatin	Orthotopic mouse models	SK-216 has predominantly anti-angiogenic mechanism, limited direct cytotoxicity reported *in vitro* in MPM; combination markedly inhibited tumor growth and prolonged survival	*In vivo*	Marked tumor inhibition and prolonged survival; synergistic enhancement	(23)
PAI-1 inhibitor + chemotherapy (oxaliplatin)	Tiplaxtinin + oxaliplatin	CRC resistant models	blocks TGF-β–induced SERPINE1 and EMT, Resensitizing resistant CRC cells	Preclinical	Strong synergy in resistant cells	(84)
PAI-1 inhibitor + chemotherapy (Doxorubicin)	Tiplaxtinin + doxorubicin	Osteosarcoma model	Combination increases cytotoxicity vs monotherapy; PAI-1 blockade prevents survival feedback and promotes apoptosis	Preclinical	Increased cytotoxicity vs monotherapy; synergy reported	(85)
PAI-1 inhibitor + chemotherapy (Paclitaxel)	TM5614 + paclitaxel	Angiosarcoma	Suppress PAI-1–mediated survival/stromal remodeling, transitional rationale strong	Early clinical exploration	Clinical outcomes pending	(86)
PAI-1 inhibitor + Radiotherapy	Tiplaxtinin + radiotherapy	NSCLC models	Blocks radiation-induced PAI-1/AKT/ERK/Snail pathway that drives EMT/radioresistance; ↓ migration/EMT/motility; ↑ apoptosis and radiosensitivity\	*In vitro*/*In vivo*	Synergy demonstrated; ↑ apoptosis, ↓ EMT/migration	(77)

Ab, antibody; AKT, protein kinase B; CAF, cancer-associated fibroblast; CRC, colorectal cancer; EMT, epithelial–mesenchymal transition; ERK, extracellular signal-regulated kinase; ESCC, esophageal squamous cell carcinoma; HUVEC, human umbilical vein endothelial cells; ICI, immune checkpoint inhibitor; MPM, malignant pleural mesothelioma; NSCLC, non-small cell lung cancer; PAI-1, plasminogen activator inhibitor-1; PD-1, programmed cell death protein 1; PI3K, phosphoinositide 3-kinase; ROS, reactive oxygen species; SERPINE1, serpin family E member 1 (gene encoding PAI-1); Snail, SNAI1 transcription factor; TGF-β, transforming growth factor beta; VEGF, vascular endothelial growth factor.

## 8 Conclusion

PAI-1 has emerged as an important regulator of tumor progression, metastasis, and treatment resistance. Beyond its classical role in fibrinolysis, it influences pericellular proteolysis, extracellular matrix remodeling, angiogenesis, and immune evasion, and elevated levels are consistently associated with poorer clinical outcomes across multiple cancers. Despite this strong biological rationale, translating these findings into clinical benefit remains challenging. Future progress will depend on clarifying the context-dependent functions of PAI-1, improving methods for measuring active PAI-1, and developing inhibitors with sufficient stability and tumor penetration while minimizing bleeding risk. In this setting, biomarker-guided patient selection and rational combination strategies will likely be necessary to determine where PAI-1 targeting can provide meaningful therapeutic benefit.

Future clinical studies should therefore integrate prospective biomarker evaluation. Parallel assessment of tumor-tissue and circulating PAI-1, together with monitoring of active PAI-1 levels over time, may provide a practical validation strategy by correlating biomarker changes with treatment response and progression patterns, enabling rational patient selection for PAI-1–targeted or combination therapies.

## References

[1] Bajou K Noël A Gerard RD Masson V Brunner N Holst-Hansen C . Absence of host plasminogen activator inhibitor 1 prevents cancer invasion and vascularization. Nat Med. (1998) 4:923–8. doi: 10.1038/nm0898-923. PMID: 9701244

[2] Kubala MH DeClerck YA . The plasminogen activator inhibitor-1 paradox in cancer: a mechanistic understanding. Cancer Metastasis Rev. (2019) 38:483–92. doi: 10.1007/s10555-019-09806-4. PMID: 31734763 PMC7001780

[3] Dass K Ahmad A Azmi AS Sarkar SH Sarkar FH . Evolving role of uPA/uPAR system in human cancers. Cancer Treat Rev. (2008) 34:122–36. doi: 10.1016/j.ctrv.2007.10.005. PMID: 18162327

[4] Mondino A Blasi F . uPA and uPAR in fibrinolysis, immunity and pathology. Trends Immunol. (2004) 25:450–5. doi: 10.1016/j.it.2004.06.004. PMID: 15275645

[5] Danø K Behrendt N Høyer-Hansen G Johnsen M Lund LR Ploug M . Plasminogen activation and cancer. Thromb Haemost. (2005) 93:676–81. doi: 10.1160/TH05-01-0054. PMID: 15841311

[6] Andreasen PA Egelund R Petersen HH . The plasminogen activation system in tumor growth, invasion, and metastasis. Cell Mol Life Sci. (2000) 57:25–40. doi: 10.1007/s000180050497. PMID: 10949579 PMC11146824

[7] Sawdey MS Loskutoff DJ . Regulation of murine type 1 plasminogen activator inhibitor gene expression *in vivo*. Tissue specificity and induction by lipopolysaccharide, tumor necrosis factor-alpha, and transforming growth factor-beta. J Clin Invest. (1991) 88:1346–53. doi: 10.1172/JCI115440. PMID: 1918385 PMC295605

[8] Yaron JR Zhang L Guo Q Haydel SE Lucas AR . Fibrinolytic serine proteases, therapeutic serpins and inflammation: Fire dancers and firestorms. Front Cardiovasc Med. (2021) 8:648947. doi: 10.3389/fcvm.2021.648947. PMID: 33869309 PMC8044766

[9] Placencio VR DeClerck YA . Plasminogen activator inhibitor-1 in cancer: Rationale and insight for future therapeutic testing. Cancer Res. (2015) 75:2969–74. doi: 10.1158/0008-5472.CAN-15-0876. PMID: 26180080 PMC4613764

[10] Ferroni P Roselli M Portarena I Formica V Riondino S La Farina F . Plasma plasminogen activator inhibitor-1 (PAI-1) levels in breast cancer - relationship with clinical outcome. Anticancer Res. (2014) 34:1153–61. 24596353

[11] Zhai B-T Tian H Sun J Zou J-B Zhang X-F Cheng J-X . Urokinase-type plasminogen activator receptor (uPAR) as a therapeutic target in cancer. J Transl Med. (2022) 20:135. doi: 10.1186/s12967-022-03329-3. PMID: 35303878 PMC8932206

[12] Binder BR Mihaly J Prager GW . uPAR-uPA-PAI-1 interactions and signaling: a vascular biologist’s view. Thromb Haemost. (2007) 97:336–42. doi: 10.1160/th06-11-0669. PMID: 17334498

[13] Olson D Pöllänen J Høyer-Hansen G Rønne E Sakaguchi K Wun TC . Internalization of the urokinase-plasminogen activator inhibitor type-1 complex is mediated by the urokinase receptor. J Biol Chem. (1992) 267:9129–33. doi: 10.1016/s0021-9258(19)50398-2 1315748

[14] Nykjaer A Conese M Christensen EI Olson D Cremona O Gliemann J . Recycling of the urokinase receptor upon internalization of the uPA:serpin complexes. EMBO J. (1997) 16:2610–20. doi: 10.1093/emboj/16.10.2610. PMID: 9184208 PMC1169872

[15] Czekay RP Kuemmel TA Orlando RA Farquhar MG . Direct binding of occupied urokinase receptor (uPAR) to LDL receptor-related protein is required for endocytosis of uPAR and regulation of cell surface urokinase activity. Mol Biol Cell. (2001) 12:1467–79. doi: 10.1091/mbc.12.5.1467. PMID: 11359936 PMC34598

[16] Wortley J Vu J Soogoor N Becerra M Sathyamoorthy M . A contemporary review of plasminogen activator inhibitor type 1: Structure, function, genetic architecture, and intracellular/extracellular roles. TH Open. (2025) 9:a26984219. doi: 10.1055/a-2698-4219. PMID: 41059382 PMC12499650

[17] McMahon GA Petitclerc E Stefansson S Smith E Wong MK Westrick RJ . Plasminogen activator inhibitor-1 regulates tumor growth and angiogenesis. J Biol Chem. (2001) 276:33964–8. doi: 10.1074/jbc.M105980200. PMID: 11441025

[18] Devy L Blacher S Grignet-Debrus C Bajou K Masson V Gerard RD . The pro‐ or antiangiogenic effect of plasminogen activator inhibitor 1 is dose dependent. FASEB J. (2002) 16:147–54. doi: 10.1096/fj.01-0552com. PMID: 11818362

[19] Stefansson S Lawrence DA . The serpin PAI-1 inhibits cell migration by blocking integrin alpha V beta 3 binding to vitronectin. Nature. (1996) 383:441–3. doi: 10.1038/383441a0. PMID: 8837777

[20] Zhou A Huntington JA Pannu NS Carrell RW Read RJ . How vitronectin binds PAI-1 to modulate fibrinolysis and cell migration. Nat Struct Biol. (2003) 10:541–4. doi: 10.1038/nsb943. PMID: 12808446

[21] Lawrence DA Palaniappan S Stefansson S Olson ST Francis-Chmura AM Shore JD . Characterization of the binding of different conformational forms of plasminogen activator inhibitor-1 to vitronectin. Implications for the regulation of pericellular proteolysis. J Biol Chem. (1997) 272:7676–80. doi: 10.1074/jbc.272.12.7676. PMID: 9065424

[22] Minor KH Peterson CB . Plasminogen activator inhibitor type 1 promotes the self-association of vitronectin into complexes exhibiting altered incorporation into the extracellular matrix. J Biol Chem. (2002) 277:10337–45. doi: 10.1074/jbc.M109564200. PMID: 11796716

[23] Takayama Y Hattori N Hamada H Masuda T Omori K Akita S . Inhibition of PAI-1 limits tumor angiogenesis regardless of angiogenic stimuli in Malignant pleural mesothelioma. Cancer Res. (2016) 76:3285–94. doi: 10.1158/0008-5472.CAN-15-1796. PMID: 27197170

[24] Lin L-L Nayak B Osmulski PA Wang E Wang C-P Valente PT . PAI-1 uncouples integrin-β1 from restrain by membrane-bound β-catenin to promote collagen fibril remodeling in obesity-related neoplasms. Cell Rep. (2024) 43:114527. doi: 10.1016/j.celrep.2024.114527. PMID: 39046873 PMC11956528

[25] Valiente M Obenauf AC Jin X Chen Q Zhang X-F Lee DJ . Serpins promote cancer cell survival and vascular co-option in brain metastasis. Cell. (2014) 156:1002–16. doi: 10.1016/j.cell.2014.01.040. PMID: 24581498 PMC3988473

[26] Schneider DJ Chen Y Sobel BE . The effect of plasminogen activator inhibitor type 1 on apoptosis. Thromb Haemost. (2008) 100:1037–40. doi: 10.1160/th08-04-0234. PMID: 19132227

[27] Webb DJ Thomas KS Gonias SL . Plasminogen activator inhibitor 1 functions as a urokinase response modifier at the level of cell signaling and thereby promotes MCF-7 cell growth. J Cell Biol. (2001) 152:741–52. doi: 10.1083/jcb.152.4.741. PMID: 11266465 PMC2195772

[28] Nguyen DH Webb DJ Catling AD Song Q Dhakephalkar A Weber MJ . Urokinase-type plasminogen activator stimulates the Ras/Extracellular signal-regulated kinase (ERK) signaling pathway and MCF-7 cell migration by a mechanism that requires focal adhesion kinase, Src, and Shc. Rapid dissociation of GRB2/Sps-Shc complex is associated with the transient phosphorylation of ERK in urokinase-treated cells. J Biol Chem. (2000) 275:19382–8. doi: 10.1074/jbc.M909575199. PMID: 10777511

[29] Maillard C Jost M Rømer MU Brunner N Houard X Lejeune A . Host plasminogen activator inhibitor-1 promotes human skin carcinoma progression in a stage-dependent manner. Neoplasia. (2005) 7:57–66. doi: 10.1593/neo.04406. PMID: 15720817 PMC1490321

[30] Nam D-E Seong HC Hahn YS . Plasminogen activator inhibitor-1 and oncogenesis in the liver disease. J Cell Signal. (2021) 2:221–7. doi: 10.33696/signaling.2.054. PMID: 34671766 PMC8525887

[31] Ohuchi K Kambayashi Y Hidaka T Fujimura T . Plasminogen activating inhibitor-1 might predict the efficacy of anti-PD1 antibody in advanced melanoma patients. Front Oncol. (2021) 11:798385. doi: 10.3389/fonc.2021.798385. PMID: 34912726 PMC8666429

[32] Miyata T . Overview: PAI-1 inhibitors and clinical applications. BioMed J. (2025) 49:100874. doi: 10.1016/j.bj.2025.100874. PMID: 40403845 PMC12859776

[33] Ibrahim AA Fujimura T Uno T Terada T Hirano K-I Hosokawa H . Plasminogen activator inhibitor-1 promotes immune evasion in tumors by facilitating the expression of programmed cell death-ligand 1. Front Immunol. (2024) 15:1365894. doi: 10.3389/fimmu.2024.1365894. PMID: 38779680 PMC11109370

[34] Tseng Y-J Lee C-H Chen W-Y Yang J-L Tzeng H-T . Inhibition of PAI-1 blocks PD-L1 endocytosis and improves the response of melanoma cells to immune checkpoint blockade. J Invest Dermatol. (2021) 141:2690–2698.e6. doi: 10.1016/j.jid.2021.03.030. PMID: 34000287

[35] Wang L Lin X Sun P . uPAR, beyond regulating physiological functions, has orchestrated roles in cancer (review). Int J Oncol. (2022) 61:151. doi: 10.3892/ijo.2022.5441. PMID: 36263620

[36] Kelly TE Spillane CL Ward MP Hokamp K Huang Y Tewari P . Plasminogen activator inhibitor 1 is associated with high-grade serous ovarian cancer metastasis and is reduced in patients who have received neoadjuvant chemotherapy. Front Cell Dev Biol. (2023) 11:1150991. doi: 10.3389/fcell.2023.1150991. PMID: 38143926 PMC10740207

[37] Wang J Peng Y Guo H Li C . PAI-1 polymorphisms have significant associations with cancer risk, especially feminine cancer. Technol Cancer Res Treat. (2021) 20:15330338211037812. doi: 10.1177/15330338211037813. PMID: 34521295 PMC8447096

[38] Masuda T Hattori N . Role of PAI-1 in the progression and treatment resistance of non-small cell lung cancer. BioMed J. (2025) 49:100911. doi: 10.1016/j.bj.2025.100911. PMID: 40915498 PMC12859764

[39] Xi X Liu N Wang Q Chu Y Yin Z Ding Y . ACT001, a novel PAI-1 inhibitor, exerts synergistic effects in combination with cisplatin by inhibiting PI3K/AKT pathway in glioma. Cell Death Dis. (2019) 10:757. doi: 10.1038/s41419-019-1986-2. PMID: 31591377 PMC6779874

[40] Roy A Coum A Marinescu VD Põlajeva J Smits A Nelander S . Glioma-derived plasminogen activator inhibitor-1 (PAI-1) regulates the recruitment of LRP1 positive mast cells. Oncotarget. (2015) 6:23647–61. doi: 10.18632/oncotarget.4640. PMID: 26164207 PMC4695142

[41] Shifman SG O’Connor JL Radin DP Sharma A Infante L Ferraresso F . Targeting autophagy and plasminogen activator inhibitor-1 increases survival and remodels the tumor microenvironment in glioblastoma. J Exp Clin Cancer Res. (2025) 44:214. doi: 10.1186/s13046-025-03473-w. PMID: 40684232 PMC12275254

[42] Jin Y Liang Z-Y Zhou W-X Zhou L . Expression, clinicopathologic and prognostic significance of plasminogen activator inhibitor 1 in hepatocellular carcinoma. Cancer biomark. (2020) 27:285–93. doi: 10.3233/CBM-190560. PMID: 31640087 PMC12662299

[43] Marquard S Thomann S Weiler SME Bissinger M Lutz T Sticht C . Yes-associated protein (YAP) induces a secretome phenotype and transcriptionally regulates plasminogen activator inhibitor-1 (PAI-1) expression in hepatocarcinogenesis. Cell Commun Signal. (2020) 18:166. doi: 10.1186/s12964-020-00634-6. PMID: 33097058 PMC7583285

[44] Sakakibara T Hibi K Koike M Fujiwara M Kodera Y Ito K . Plasminogen activator inhibitor-1 as a potential marker for the Malignancy of colorectal cancer. Br J Cancer. (2005) 93:799–803. doi: 10.1038/sj.bjc.6602743. PMID: 16091756 PMC2361636

[45] Bal O Ekinci AS Dogan M Atay C Demirci A Oksuzoglu B . The prognostic and predictive significance of plasma type 1 plasminogen activator inhibitor and endoglin in metastatic colorectal cancer patients treated with bevacizumab-containing chemotherapy. J Cancer Res Ther. (2019) 15:48–53. doi: 10.4103/jcrt.JCRT_1253_16. PMID: 30880754

[46] Huang C-F Yu G-T Wang W-M Liu B Sun Z-J . Prognostic and predictive values of SPP1, PAI and caveolin-1 in patients with oral squamous cell carcinoma. Int J Clin Exp Pathol. (2014) 7:6032–9. PMC4203219 25337248

[47] Sillen M Declerck PJ . A narrative review on plasminogen activator inhibitor-1 and its (patho)physiological role: To target or not to target? Int J Mol Sci. (2021) 22:2721. doi: 10.3390/ijms22052721. PMID: 33800359 PMC7962805

[48] Elokdah H Abou-Gharbia M Hennan JK McFarlane G Mugford CP Krishnamurthy G . Tiplaxtinin, a novel, orally efficacious inhibitor of plasminogen activator inhibitor-1: design, synthesis, and preclinical characterization. J Med Chem. (2004) 47:3491–4. doi: 10.1021/jm049766q. PMID: 15214776

[49] Jeong BY Uddin MJ Park JH Lee JH Lee HB Miyata T . Novel plasminogen activator inhibitor-1 inhibitors prevent diabetic kidney injury in a mouse model. PLoS One. (2016) 11:e0157012. doi: 10.1371/journal.pone.0157012. PMID: 27258009 PMC4892642

[50] Placencio VR Ichimura A Miyata T DeClerck YA . Small molecule inhibitors of plasminogen activator inhibitor-1 elicit anti-tumorigenic and anti-angiogenic activity. PLoS One. (2015) 10:e0133786. doi: 10.1371/journal.pone.0133786. PMID: 26207899 PMC4514594

[51] Mashiko S Kitatani K Toyoshima M Ichimura A Dan T Usui T . Inhibition of plasminogen activator inhibitor-1 is a potential therapeutic strategy in ovarian cancer. Cancer Biol Ther. (2015) 16:253–60. doi: 10.1080/15384047.2014.1001271. PMID: 25587663 PMC4623014

[52] Hendrikson J Liu Y Ng WH Lee JY Lim AH Loh JW . Ligand-mediated PAI-1 inhibition in a mouse model of peritoneal carcinomatosis. Cell Rep Med. (2022) 3:100526. doi: 10.1016/j.xcrm.2022.100526. PMID: 35243423 PMC8861959

[53] Zheng Y Meng L Qu L Zhao C Wang L Liu C . Anti-PAI-1 monoclonal antibody inhibits the metastasis and growth of esophageal squamous cell carcinoma. J Cancer. (2023) 14:114–28. doi: 10.7150/jca.77888. PMID: 36605486 PMC9809335

[54] Vaughan DE . PAI-1 and atherothrombosis. J Thromb Haemost. (2005) 3:1879–83. doi: 10.1111/j.1538-7836.2005.01420.x. PMID: 16102055

[55] Lawrence DA Olson ST Palaniappan S Ginsburg D . Engineering plasminogen activator inhibitor 1 mutants with increased functional stability. Biochemistry. (1994) 33:3643–8. doi: 10.1021/bi00178a022. PMID: 8142362

[56] Lawrence DA Olson ST Palaniappan S Ginsburg D . Serpin reactive center loop mobility is required for inhibitor function but not for enzyme recognition. J Biol Chem. (1994) 269:27657–62. doi: 10.1016/s0021-9258(18)47036-6 7961684

[57] Lawrence DA Ginsburg D Day DE Berkenpas MB Verhamme IM Kvassman JO . Serpin-protease complexes are trapped as stable acyl-enzyme intermediates. J Biol Chem. (1995) 270:25309–12. doi: 10.1074/jbc.270.43.25309. PMID: 7592687

[58] Shang L Xue G Gong L Zhang Y Peng S Yuan C . A novel ELISA for the detection of active form of plasminogen activator inhibitor-1 based on a highly specific trapping agent. Anal Chim Acta. (2019) 1053:98–104. doi: 10.1016/j.aca.2018.12.005. PMID: 30712574

[59] Scheer FAJL Shea SA . Human circadian system causes a morning peak in prothrombotic plasminogen activator inhibitor-1 (PAI-1) independent of the sleep/wake cycle. Blood. (2014) 123:590–3. doi: 10.1182/blood-2013-07-517060. PMID: 24200683 PMC3901072

[60] Francis RM Romeyn CL Coughlin AM Nagelkirk PR Womack CJ Lemmer JT . Age and aerobic training status effects on plasma and skeletal muscle tPA and PAI-1. Eur J Appl Physiol. (2014) 114:1229–38. doi: 10.1007/s00421-014-2857-2. PMID: 24604072

[61] Li S Wei X He J Tian X Yuan S Sun L . Plasminogen activator inhibitor-1 in cancer research. BioMed Pharmacother. (2018) 105:83–94. doi: 10.1016/j.biopha.2018.05.119. PMID: 29852393

[62] Masuda T Hirata T Sakamoto T Tsubata Y Ichihara E Kozuki T . Treatment rationale and protocol design: an investigator-initiated phase II study of combination treatment of nivolumab and TM5614, a PAI-1 inhibitor for previously treated patients with non-small cell lung cancer. J Thorac Dis. (2024) 16:3381–8. doi: 10.21037/jtd-23-1858. PMID: 38883673 PMC11170418

[63] Fujimura T Yoshino K Kato H Fukushima S Ishizuki S Otsuka A . A phase II multicentre study of plasminogen activator inhibitor-1 inhibitor (TM5614) plus nivolumab for treating anti-programmed cell death 1 antibody-refractory Malignant melanoma: TM5614-MM trial. Br J Dermatol. (2024) 191:691–7. doi: 10.1093/bjd/ljae231. PMID: 38833158

[64] Hirai T Asano K Ito I Miyazaki Y Sugiura H Agirbasli M . A randomized double-blind placebo-controlled trial of an inhibitor of plasminogen activator inhibitor-1 (TM5614) in mild to moderate COVID-19. Sci Rep. (2024) 14:165. doi: 10.1038/s41598-023-50445-1. PMID: 38168544 PMC10761996

[65] Shimpi A Juvale K . A comprehensive review on the role of acetamido as a linker for the design and discovery of anticancer agents. Med Oncol. (2025) 42:496. doi: 10.1007/s12032-025-03043-2. PMID: 41003852

[66] Sotiropoulos G Kotopouli M Karampela I Christodoulatos GS Antonakos G Marinou I . Circulating plasminogen activator inhibitor-1 activity: a biomarker for resectable non-small cell lung cancer? J Buon. (2019) 24:943–54. 31424646

[67] Chen JJ Vincent MY Shepard D Peereboom D Mahalingam D Battiste J . Phase 1 dose expansion and biomarker study assessing first-in-class tumor microenvironment modulator VT1021 in patients with advanced solid tumors. Commun Med. (2024) 4:95. doi: 10.1038/s43856-024-00520-z. PMID: 38773224 PMC11109328

[68] Lu J-J Guo H Gao B Zhang Y Lin Q-L Shi J . Prognostic value of urokinase plasminogen activator system in non-small cell lung cancer: a systematic review and meta-analysis. Mol Clin Oncol. (2018) 8:127–32. doi: 10.3892/mco.2017.1484. PMID: 29387404 PMC5769288

[69] Zekanowska E Cieśliński K Rość D . Plasminogen activator inhibitor type 1 (PAI-1) in blood and tissue extracts of patients with non-small cell lung cancer. Pneumonol Alergol Pol. (2004) 72:409–14. 16021996

[70] Ye F Wu X Jeong E-K Jia Z Yang T Parker D . A peptide targeted contrast agent specific to fibrin-fibronectin complexes for cancer molecular imaging with MRI. Bioconjug Chem. (2008) 19:2300–3. doi: 10.1021/bc800211r. PMID: 19053180 PMC2651601

[71] Cheng Z Jin Y Li J Shi G Yu L Shao B . Fibronectin-targeting and metalloproteinase-activatable smart imaging probe for fluorescence imaging and image-guided surgery of breast cancer. J Nanobiotechnology. (2023) 21:112. doi: 10.1186/s12951-023-01868-5. PMID: 36978072 PMC10053476

[72] Furuya H Sasaki Y Chen R Peres R Hokutan K Murakami K . PAI-1 is a potential transcriptional silencer that supports bladder cancer cell activity. Sci Rep. (2022) 12:12186. doi: 10.1038/s41598-022-16518-3. PMID: 35842542 PMC9288475

[73] Ayoub NM . Editorial: Novel combination therapies for the treatment of solid cancers. Front Oncol. (2021) 11:708943. doi: 10.3389/fonc.2021.708943. PMID: 34222030 PMC8250861

[74] Tan S Day D Nicholls SJ Segelov E . Immune checkpoint inhibitor therapy in oncology: current uses and future directions: JACC: CardioOncology state-of-the-art review. JACC CardioOncol. (2022) 4:579–97. doi: 10.1016/j.jaccao.2022.09.004. PMID: 36636451 PMC9830229

[75] Mathews SG Krishna RBD M L K N Murali S Agarwal P . The role of the plasminogen activator inhibitor 1 (PAI1) in ovarian cancer: mechanisms and therapeutic implications. Glob Med Genet. (2024) 11:358–65. doi: 10.1055/s-0044-1791734. PMID: 39583124 PMC11521755

[76] Che Y Wang J Li Y Lu Z Huang J Sun S . Cisplatin-activated PAI-1 secretion in the cancer-associated fibroblasts with paracrine effects promoting esophageal squamous cell carcinoma progression and causing chemoresistance. Cell Death Dis. (2018) 9:759. doi: 10.1038/s41419-018-0808-2. PMID: 29988148 PMC6037765

[77] Kang J Kim W Kwon T Youn H Kim JS Youn B . Plasminogen activator inhibitor-1 enhances radioresistance and aggressiveness of non-small cell lung cancer cells. Oncotarget. (2016) 7:23961–74. doi: 10.18632/oncotarget.8208. PMID: 27004408 PMC5029677

[78] Schilling D Bayer C Geurts-Moespot A Sweep FCGJ Pruschy M Mengele K . Induction of plasminogen activator inhibitor type-1 (PAI-1) by hypoxia and irradiation in human head and neck carcinoma cell lines. BMC Cancer. (2007) 7:143. doi: 10.1186/1471-2407-7-143. PMID: 17663760 PMC1973081

[79] Jänicke F Schmitt M Pache L Ulm K Harbeck N Höfler H . Urokinase (uPA) and its inhibitor PAI-1 are strong and independent prognostic factors in node-negative breast cancer. Breast Cancer Res Treat. (1993) 24:195–208. doi: 10.1007/BF01833260. PMID: 8435475

[80] Gorlatova NV Cale JM Elokdah H Li D Fan K Warnock M . Mechanism of inactivation of plasminogen activator inhibitor-1 by a small molecule inhibitor. J Biol Chem. (2007) 282:9288–96. doi: 10.1074/jbc.M611642200. PMID: 17276980

[81] Noguchi R Kaji K Namisaki T Moriya K Kawaratani H Kitade M . Novel oral plasminogen activator inhibitor-1 inhibitor TM5275 attenuates hepatic fibrosis under metabolic syndrome via suppression of activated hepatic stellate cells in rats. Mol Med Rep. (2020) 22:2948–56. doi: 10.3892/mmr.2020.11360. PMID: 32945412 PMC7453658

[82] Banik SM Pedram K Wisnovsky S Ahn G Riley NM Bertozzi CR . Lysosome-targeting chimaeras for degradation of extracellular proteins. Nature. (2020) 584:291–7. doi: 10.1038/s41586-020-2545-9. PMID: 32728216 PMC7727926

[83] Lin J Jin J Shen Y Zhang L Gong G Bian H . Emerging protein degradation strategies: expanding the scope to extracellular and membrane proteins. Theranostics. (2021) 11:8337–49. doi: 10.7150/thno.62686. PMID: 34373745 PMC8344007

[84] Wong SQR Das M Tenzin K Shirgaonkar N Chua H Chee LX . Modeling oxaliplatin resistance in colorectal cancer reveals a SERPINE1-based gene signature (RESIST-M) and therapeutic strategies for pro-metastatic CMS4 subtype. Cell Death Dis. (2025) 16:529. doi: 10.1038/s41419-025-07855-y. PMID: 40664638 PMC12264272

[85] Cho Y-E Kim S-C Kim HJ Han I Ku J-L . Establishment and characterization of 18 sarcoma cell lines: unraveling the molecular mechanisms of doxorubicin resistance in sarcoma cell lines. J Transl Med. (2024) 22:889. doi: 10.1186/s12967-024-05700-y. PMID: 39358756 PMC11445991

[86] Fujimura T Yoshino K Nakamura M Kato H Ito T Maekawa T . Efficacy and safety of TM5614 in combination with paclitaxel in the treatment of paclitaxel-resistant cutaneous angiosarcoma: phase II study protocol. Exp Dermatol. (2024) 33:e14976. doi: 10.1111/exd.14976. PMID: 37946551

